# siRNA could be a potential therapy for COVID-19 

**DOI:** 10.17179/excli2020-1328

**Published:** 2020-04-22

**Authors:** Sanhita Ghosh, Sayeed Mohammad Firdous, Anirban Nath

**Affiliations:** 1 Department of Genetics and Plant Breeding, Institute of Agricultural Science, University of Calcutta, 51/2, Hazra Road, Kolkata 700073, West Bengal, India; 2 Department of Pharmacology, Calcutta Institute of Pharmaceutical Technology & AHS, Uluberia, Howrah 711316, West Bengal, India

## ⁯⁯

***Dear Editor,***

Small interfering RNA (siRNA), sometimes known as short interfering RNA or silencing RNA, is a class of double-stranded non-coding RNA molecules, which is 20-25 base pairs in length. siRNAs can regulate the expression of genes, by a phenomenon known as RNA interference (RNAi). Based on the phenomenon, the siRNA based therapeutics have been developed and implemented for anticancer, antiviral, and genetic diseases (Liu et al*.*, 2020[4]).

In December 2019, WHO reported the outbreak of a novel coronavirus, designated as SARS-CoV-2 or severe acute respiratory syndrome-related coronavirus. This virus has currently spread across 212 countries which resulted in 2,416,135 active cases of infection, and approximately 165,939 mortalities, as per WHO (2020[9]). There are many drugs currently being tested which include antiviral (remdesivir, favipiravir, lopinavir, ritonavir, and arbidol), anti-malarial (hydroxychloroquine), and anticancer (interferon-alpha 2b) agents. These drug candidates are undergoing clinical trials, and their efficacy against SARS-CoV-2 has yet to be proven. Under such a situation, siRNA based treatment can provide an effective solution in combating COVID-19 (Liu et al**.**, 2020[4]). Some earlier studies revealed that siRNA candidates were effectively used against the outbreak of SARS and Middle-East Respiratory Syndrome (MERS), recapitulated in Table 1(Tab. 1) (References in Table 1: Weimin H, Li S, Aili L, 2003[8]; Tang QQ, Lu PY, Xie FY, Liu Y, Xu J, Woodle MC, 2004[6]; Wang Y, Liu L, Wang S, Zhang Y, 2006[7]; Zhang Y, Wang G, Li M, Wang H, Feng H, 2006[12]; Sun B, Zheng B, Lv W, Xu K, 2006[5]; Elmén J, Wahlestedt C, Liang Z, Sørensen MA, Ørum H, Koch T, 2014[2]).

The siRNAs identified successfully targeted the sequences which coded for the viral RNA-dependent RNA polymerase, helicase, proteolytic enzymes, and the nucleoprotein N of earlier SARS virus leading to a 50, 70, 90, and 95 % decrease in viral load, respectively. The viral genome of SARS-CoV-2 is 29 kbp in size and one of the largest genomes among the RNA virus. This genome consists of fourteen open reading frames (ORFs) which coded for twenty-seven structural and nonstructural proteins (Wu et al., 2020[10]). At the 5' end, there are the two largest ORFs, namely ORF1a and ORF1b which are translated into a single large poly-protein by the ribosome through a frame-shift event. The ORF1a comprises of two viral cysteine proteases, namely papain-like protease (nsp3) and the main protease designated as 3-chymotrypsin-like protease or 3CL. Among two viral proteases the sequence which codes for the nsp3 has been reported to be less conserved (Liu et al., 2020[4]). However, the sequence which codes for the protease 3CL (nsp5) has been observed to be highly conserved among the annotated sequences (Wu et al., 2020[11]). Currently, the protease has been considered as a major drug target for multiple antiviral agents, which are presently undergoing clinical trials. Thus, the sequence coding for nsp5 can be treated as a potential target for RNAi using siRNA based therapeutics. Other potential targets include the viral RNA dependent RNA polymerase (Rd-Rp) which is located in the ORF1b, stretching from 13-16 kbp on the viral genome. Downstream to the sequences (from 16-18 kbp) the site coding for the viral helicase has been identified. These two sites have been reported to be highly conserved among the annotated genome of SARS-CoV-2 along with the earlier genomes of beta coronaviruses like SARS and MERS (Wu et al., 2020[11]). Thus, these two sites can be considered to be potential targets for RNAi using siRNA.

To date, various delivery systems for siRNA have been identified which are broadly classified into nanoparticles based carriers and viral vectors. In 2003, siRNA based drug was developed by Sirnaomics, Inc. (Maryland, USA) for the outbreak of SARS-CoV and H5N1 influenza. Besides, in 2017, six siRNA have been developed by Alnylam Pharmaceuticals (USA) and Vir Biotechnology against infectious diseases. Further, Alnylam Pharmaceuticals (USA) has designed and synthesized over 350 siRNA targeting highly conserved regions of the available SARS-CoV-2 genome (Hodgson, 2020[3]). However, effective carriers must be identified for the successful delivery of the drug payload at the areas which are predominantly infected by the pathogen.

For SARS-CoV-2, the ciliated cells of the human lungs are the primary site for viral infection, with reports indicating viral transmission via contact, droplets of saliva or fomites from the infected person. Therefore, techniques designed for optimal delivery of drugs onto the lung epithelial cells can provide better and timely results. In this context, Conti and co-researchers have demonstrated an *in vitro* testing of poly (amidoamine) dendrimer nanocarriers for the potential aerosol-based delivery system of siRNA onto lung epithelial cells (Conti et al., 2014[1]). However, the nanocarrier delivery system has limits to its efficient delivery. Hence, there is a need to overcome these limitations by formulating an effective delivery system that can offer unique advances to the field of inhaled siRNA formulation.

Despite the pandemic outbreaks of COVID-19 and the high rate of transmission in humans, there is no specific treatment for the COVID-19 at present. Thus, for the treatment of COVID-19 siRNA based therapy can be developed against the novel coronavirus SARS-CoV-2, where siRNAs can hit the highly conserved region of SARS-CoV-2 RNA and also can act as an inhibitor to suppress the genetic disorders of the lungs. This approach could help to achieve a better treatment goal that can reduce the pandemic threat of COVID-19.

## Conflict of interest

The authors declare no conflict of interest.

## Figures and Tables

**Table 1:**
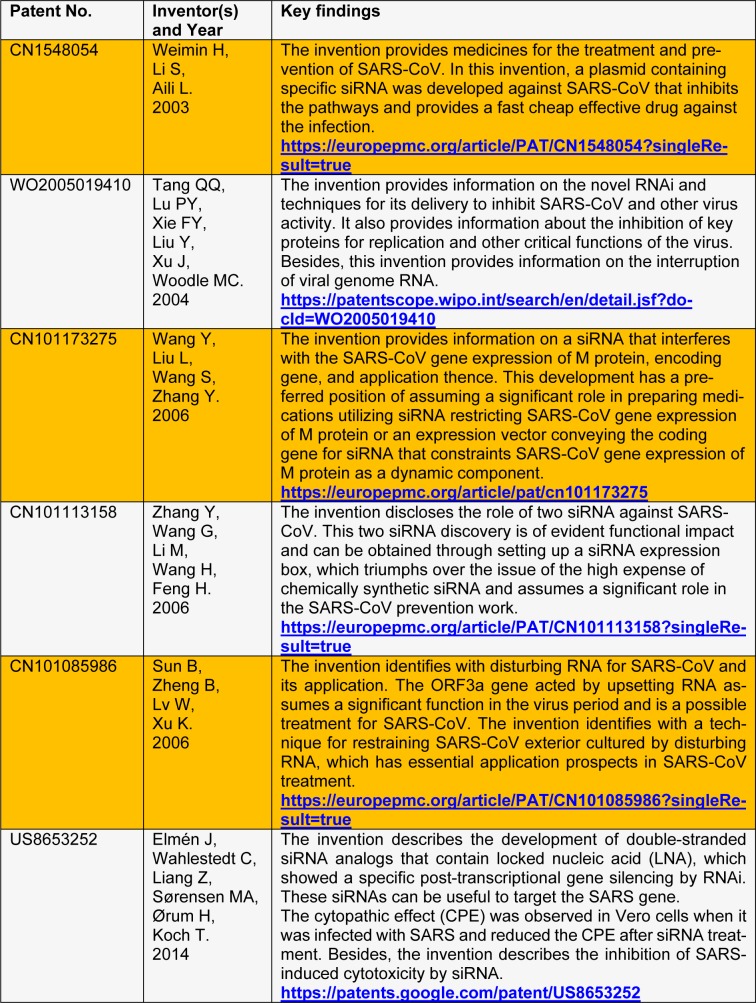
List of some siRNA related patents associated with coronaviruses
